# Biomimetic bimodal haptic perception using triboelectric effect

**DOI:** 10.1126/sciadv.ado6793

**Published:** 2024-07-05

**Authors:** Shaoshuai He, Jinhong Dai, Dong Wan, Shengshu Sun, Xiya Yang, Xin Xia, Yunlong Zi

**Affiliations:** ^1^ Thrust of Sustainable Energy and Environment, The Hong Kong University of Science and Technology (Guangzhou), Nansha, Guangzhou 511400, Guangdong, China.; ^2^ Medical School, Chinese PLA, Fuxing Road 28, Beijing 100853, China.; ^3^ Institute of New Energy Technology, College of Physics & Optoelectronic Engineering, Jinan University, Guangzhou 510632, Guangdong, China.; ^4^ HKUST Shenzhen-Hong Kong Collaborative Innovation Research Institute, Futian, Shenzhen 518057, Guangdong, China.; ^5^ Guangzhou HKUST Fok Ying Tung Research Institute, Nansha, Guangzhou 511400, Guangdong, China.

## Abstract

Multimodal haptic perception is essential for enhancing perceptual experiences in augmented reality applications. To date, several artificial tactile interfaces have been developed to perceive pressure and precontact signals, while simultaneously detecting object type and softness with quantified modulus still remains challenging. Here, inspired by the campaniform sensilla on insect antennae, we proposed a hemispherical bimodal intelligent tactile sensor (BITS) array using the triboelectric effect. The system is capable of softness identification, modulus quantification, and material type recognition. In principle, due to the varied deformability of materials, the BITS generates unique triboelectric output fingerprints when in contact with the tested object. Furthermore, owing to the different electron affinities, the BITS array can accurately recognize material type (99.4% accuracy), facilitating softness recognition (100% accuracy) and modulus quantification. It is promising that the BITS based on the triboelectric effect has the potential to be miniaturized to provide real-time accurate haptic information as an artificial antenna toward applications of human-machine integration.

## Teaser

The bimodal haptic sensor can recognize material type and softness and quantify Young’s modulus using the triboelectric effect.

## INTRODUCTION

Tactile perception plays a vital role in how humans gather information and receive feedback about their surroundings. With the advent of the “Artificial Intelligence” era, state-of-the-art intelligent robots endowed with human-like sensory capabilities have garnered increasing attention. Drawing inspiration from bioreceptors in biological organisms, tactile sensors that exhibit high responsiveness to external physical stimuli [e.g., pressure (*1*, *2*), temperature (*3*), and humidity (*4*)] in the environment have demonstrated applications in a wide range of bionic electronics, including but not limited to aircraft, humanoid robots, exploration robots, prosthetics, and actuators (*5*–*8*). However, despite their capability to perceive precontact stimuli and pressure magnitude, these sensors failed to differentiate object types, assess softness, and quantify Young’s modulus magnitude (*9*–*11*). This capability is essential for facilitating interaction between robots and their environment, especially in tasks like navigation, obstacle avoidance, environmental exploration, and tactile feedback delivery in prosthetics. Meanwhile, the digitization of tactile recognition enables objective and quantitative perception, greatly expanding the range and depth of tactile information acquisition for intelligent robots and medical rehabilitation. Therefore, it is paramount to develop multimodal sensors capable of simultaneously identifying the material type and softness.

Considerable efforts have been dedicated to developing tactile sensors to perceive object type and deformability using various strategies. Material type identification can be accomplished using computer vision, thermal conductivity, triboelectric effect, etc. (*12*, *13*). In addition, tactile sensors with sophisticated structures have been designed to detect object deformability. However, these technologies still have limitations in accurately identifying the material type and quantifying softness. Although triboelectric sensor arrays can detect the type of material, it is challenging to quantify the material’s softness (*14*, *15*), which refers to the deformability of the material under pressure and is related to Young’s modulus of the material (fig. S1). By applying contact mechanics theories, such as the Hertz model, it becomes possible to calculate Young’s modulus by analyzing the relationship between force and displacement during the indentation process, where pressure is exerted on the sample using an indenter (*16*–*19*). On the basis of the Hertz model, a self-locking strain transducer was designed to estimate Young’s modulus of materials (*20*). However, the intricate self-locked structure of the strain sensor requires a predetermined displacement to detect the sensor signal, limiting its applicability in diverse scenarios. The sensor was further simplified to be a freestanding structure at the cost of quantitative accuracy (*21*). Inspired by finger mechanoreceptors, a bionic finger with dual-layered pressure sensors was developed to detect the deformability of unstructured surfaces (*22*). However, the bionic finger can only provide a relative softness of the object and cannot measure the exact Young’s modulus, limiting its application in accurate modulus measurements, such as exploration robots and medical monitoring devices. Moreover, as artificial antennae (*23*), autonomous robots (*24*, *25*), and insect-scale robots (*26*) flourish, there is a growing demand for softness sensors that are compact in size and can be easily integrated. Therefore, the primary challenge lies in the absence of an easily integrable tactile sensor that can simultaneously provide information on material types, softness, and quantifiable Young’s modulus, enabling the sensor to perceive multiple relevant contact variables in complex environments.

**Fig. 1.**
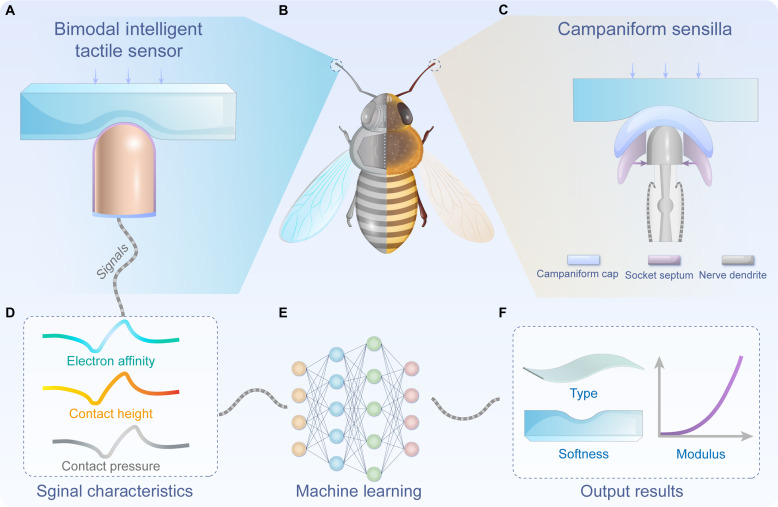
Schematic illustration of the bioinspired BITS. (**A**) Illustration of the BITS contacts an elastomer. Illustration of (**B**) the insect antenna and (**C**) the CS on the antenna for bimodal electric signals of displacement and pressure detection. (**D**) Signals (electron affinity, contact height, and contact pressure) perceived by the BITS for (**E**) machine learning to (**F**) identify material type and softness and quantify modulus.

In nature, insects use antennae to perceive changes in complicated surrounding environments (*27*). Unlike mammalian whiskers, antennae are equipped with a diverse array of sensory receptors in microscale size, including those for touch, temperature, and humidity. Among these sensory receptors, the campaniform sensilla (CS) serves as the tactile receptor, having the remarkable ability to detect and distinguish bimodal electric signals of indentation displacement and corresponding pressure caused by contact and lateral compression of the nerves (Fig. 1, B and C) (*28*–*30*). Moreover, the CS exhibits a small size and is extensively distributed within the antennae and throughout the entire body of insects. This widespread presence enables insects to detect and respond to variations in the changeable environment and interpret contact clues.

Drawing inspiration from the anatomical structure of the CS, we have developed a bimodal intelligent tactile sensor (BITS) using the triboelectric effect. The BITS adopts a single-electrode configuration consisting of a hemispherical electrode and a polymer triboelectric layer, enabling it to identify material types, recognize softness, and quantify modulus accurately (Fig. 1A). In addition, a pressure sensor is used to measure the contact force. On the basis of the triboelectric effect, the triboelectric open-circuit voltage (*V*_OC_) increases with the contact area. Therefore, by applying the Hertz model, the BITS has the potential to calculate the exact value of Young’s modulus by detecting contact area and contact pressure simultaneously. Moreover, with the triboelectric signals generated during contact with a tested material, the BITS array with various triboelectric layers can determine the material type assisted by machine learning algorithms based on its position in the triboelectric series (Fig. 1, D to F) (*31*). Therefore, this work demonstrates a universal strategy enabled by the triboelectric effect to detect the material type and softness as reflected by modulus, which may have a wide application in wearable electronics, exploration robots, intelligent prosthetics, augmented reality, etc.

## RESULTS

To provide a haptic perception of material type and modulus, we developed a BITS with a hemispherical structure inspired by the CS on insect antennas. The device uses polymer materials as the triboelectric layer and hemispherical electrode pillars as the electrodes. The BITS collects contact height, pressure, and electron affinity information to identify material type and softness during multimodal haptic perception tasks.

### System design and working mechanism

**Fig. 2.**
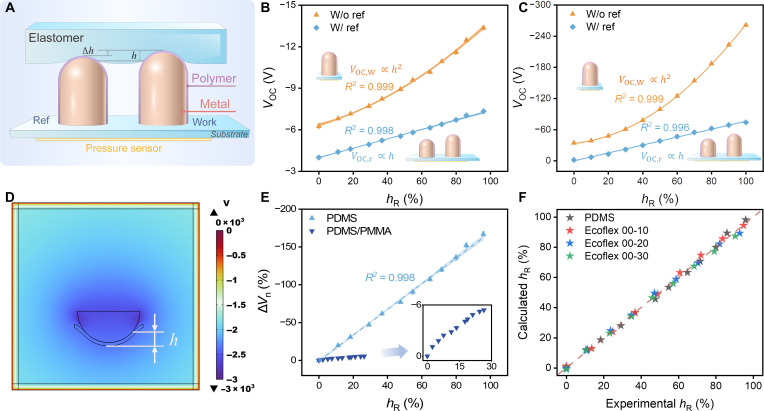
Relationships between open-circuit voltage *V*_OC_ and normalized contact height *h*_R_. (**A**) Illustration of BITS units contacting an elastomer. (**B**) Experiment results and (**C**) COMSOL simulation results of the relationship between open-circuit voltage *V*_OC_ and *h*_R_ with and without a reference electrode. (**D**) COMSOL simulation (*V*_OC_ at 15% *h*_R_). (**E**) Δ*V*_n_ versus *h*_R_ when contacting with PDMS elastomer and PDMS/PMMA. (**F**) Consistency between the calculated *h*_R_ and experimental *h*_R_ regarding PDMS elastomer, Ecoflex 00-10, Ecoflex 00-20, and Ecoflex 00-30.

Rapid and realistic haptic perception involving material type and softness information is essential for conveying multimodal sensory information about the environment. At its essence, conventional tactile sensors based on pressure detection (fig. S2) can only reflect external pressing forces without material softness information, impeding the development of multimodal somatosensory. Inspired by the CS structure on insect antennae, the BITS was designed with a hemispherical structure to collect bimodal signals related to material type and contact height, which depended on the material’s modulus and contact force. Here, the contact height *h* is defined as the height of the spherical crown in contact with the elastomer, as illustrated in Fig. 2A. When a hemispherical indenter contacts a compressible material, the contact height increases as the force increases and decreases as the material modulus increases. The finger perceives the softness of a material by a similar principle (*32*). This relationship can be well described by the Hertz contact model (Eq. 1; see details in text S1 and fig. S3), which reveals the relationship between the contact force *F*, the contact height *h*, and the material modulus *E*_s_ during the contact process

$$E_{s}=\frac{3}{4}\mathrm{FR}\left(1-v_{s}^{2}\right){\left(2\mathrm{Rh}-h^{2}\right)}^{-\frac{3}{2}}$$ (1)

where *R* is the spherical indenter radius, and *v*_s_ is the Poisson’s ratio of the material. On the basis of the Hertz model, the material modulus can be derived from contact force *F* and contact height *h* because the indenter radius *R* is known, and Poisson’s ratio *v*_s_ value can be determined based on the material type through various methods and is regarded as a known parameter (text S1). Therefore, to calculate the sample’s modulus, the contact height *h* becomes the primary parameter to be determined.

The triboelectric effect involves electron transfer between two materials at their contact interfaces, which exists in most material pairs (*14*, *33*). A triboelectric sensor in single-electrode mode was used to elaborate the working mechanism with fluorinated ethylene propylene (FEP) as the triboelectric layer and nitrile butadiene rubber (NBR) as the test sample (fig. S4). Fundamentally, the process involves triboelectrification and electrostatic induction and can be separated into four steps. In the initial state, the NBR was far from the FEP, with no signal generated in the charge-balanced state. Then, when the NBR approached the sensor, the positive charges induced on the copper electrode decreased, and the electrons flowed from the reference electrode to the copper electrode. The voltage potential difference between the copper electrode and FEP reached a maximum value when the NBR fully contacted the sensor. Last, the sensor was gradually separated from the NBR sample. During the separation process, the negatively charged FEP induced positive charges on the copper, and the electrons flowed back to the reference electrode, accompanied by a reversed output signal. The charges recovered to the initial status when the NBR was completely separated from the FEP.

The open-circuit voltage of the triboelectric nanogenerator (TENG) is related to the contact area (*34*, *35*). For a BITS with a hemispherical structure, there is a linear relationship between the contact area *S* and the contact height *h* during the contacting process, as described by Eq. 2 (see details in text S1)

S=2πRh (2)

By establishing the relationship with open-circuit voltage *V*_OC_, contact height *h* can be readily determined for quantifying material modulus based on the Hertz model (Eq. 1). During the contacting process, the open-circuit voltage of the working electrode *V*_OC,W_ can be derived (see details in text S2) regarding the contact process when the contact-separation distance is infinitely small

$$V_{\text{OC},W}=\frac{\sigma \pi h^{2}}{C}+\delta V$$ (3)

in which *C* is the capacitance of the TENG, σ is the surface charge density of the triboelectric layer, and δ*V* is the voltage variations caused by capacitance. The *V*_OC,W_ presents a quadratic relationship to *h*, mainly because the contact area changes quadratically to *h* during the contact process. However, when there is a reference electrode with a lower height Δ*h*, the relative open-circuit voltage *V*_OC,r_ (defined as the voltage difference between working and reference electrodes) presents a linear relationship to contact height *h*, as shown in the following equation (see text S2 for derivations)

$$V_{\text{OC},r}=\frac{\pi \sigma }{C}\left(2h∆h-∆h^{2}\right)$$ (4)

The reference electrode minimizes environmental influences on the open-circuit voltage and transforms the nonlinear relationship into the linear one, which is beneficial for detecting material softness and accurately quantifying material modulus. Considering the contact-separation distance, the *V*_OC,r_ can be obtained when the surfaces approach each other

$$V_{\text{OC},r}=\frac{\sigma \pi R^{2}}{C_{a}}+\frac{\sigma \sigma }{C}\left(2h∆h-∆h^{2}\right)$$ (5)

*C*_a_ is the capacitance of TENG during the approaching process. Meanwhile, the electron affinity of the test sample reflected by the charge density σ of the BITS with different triboelectric layers is harnessed for material type identification. On the basis of the mechanism, materials with different electron affinities and deformabilities were used to investigate the relationship between open-circuit voltage and contact height using the BITS. Furthermore, pressure sensors attached to the BITS were used to measure the magnitude of contact pressure.

To demonstrate the theoretical relationship between the open-circuit voltage *V*_OC_ and contact height *h*, experiments and simulations were conducted using polydimethylsiloxane (PDMS) as the test material and FEP BITS (Fig. 2A). Normalized contact height *h*_R_ (defined as the ratio of the measured contact height *h* over the indenter radius *R*) was used to investigate the relationship in the following contents. When the FEP surface contacted PDMS, the open-circuit voltage of the working electrode *V*_OC,W_ increased in proportion to the quadratic relationship with the normalized contact height *h*_R_, satisfying the theoretical relationship well (Eq. 3, Fig. 2B, and fig. S5A). To eliminate the impact of environmental factors, another BITS with a 1-mm Δ*h* was introduced as the reference, and the relative output voltage *V*_OC,r_ between the higher and lower BITSs was recorded. Under this circumstance, the *V*_OC,r_ was demonstrated to be proportional to *h*_R_ (Fig. 2B and fig. S5B), consistent with Eq. 5 well with a high *R*^2^ = 0.998, confirming the linear relationship. In addition, the finite element analysis (FEA) was conducted to investigate the relationship between the open-circuit voltages and *h*_R_ using the electrostatic and solid mechanics modules of COMSOL Multiphysics (Fig. 2, C and D). The simulation results were consistent with the experimental results. In addition to the open-circuit voltage, the short-circuit charge (*Q*_sc_) and short-circuit current (*I*_sc_) showed the linear relationship with *h*_R_ (fig. S6), facilitating the calculation of the Young’s modulus. Thus, the reference electrode was used in the following experiments to realize a better sensing functionality.

To assess the capability of softness identification, we conducted tests on samples with different softness using the BITS. Here, the normalized open-circuit voltage change (Δ*V*_n_) is defined as the ratio of voltage variation over the initial *V*_OC_ and used to identify softness and quantify modulus

$$∆V_{n}=\frac{V_{h}-V_{0}}{|V_{0}|}=\frac{\frac{2∆h}{c}}{|\frac{R^{2}}{C_{a}}-\frac{∆h^{2}}{C}|}h\times 100\%$$ (6)

where *V_h_* is the open-circuit voltage at a specific contact height, and *V*_0_ is the open-circuit voltage when the contact height is zero. When contacting pure PDMS elastomer and PDMS coated on a relative hard substrate of poly(methyl methacrylate) (PMMA) (with Young’s modulus of above 3 GPa), which are named PDMS and PDMS/PMMA, respectively, distinct signal characteristics were observed (Fig. 2E). The compressible PDMS exhibited an increase in Δ*V*_n_ as contact height *h*_R_ increased, with a good linear relationship of *R*^2^ = 0.998. In contrast, the PDMS/PMMA lacked deformability, resulting in a relatively slow increase in Δ*V*_n_ as contact height increased. Therefore, the BITS can readily obtain the material’s softness information by detecting Δ*V*_n_. Furthermore, the *V*_OC_ of PDMS showed a clear increase/decrease as force increases/decreases (fig. S7A). On the other hand, the *V*_OC_ of PDMS/PMMA only altered when the indenter contacted or detached, showing no noticeable Δ*V*_n_ with changes in force (fig. S7B). Moreover, the BITS can measure contact height precisely. The good consistency between the contact height *h*_R_ calculated by Δ*V*_n_ and that measured by the linear motor using PDMS elastomer, Ecoflex 00-10, Ecoflex 00-20, and Ecoflex 00-30 as samples demonstrated the accuracy of the BITS (Fig. 2F).

### Characterization of the softness haptic perception system

**Fig. 3.**
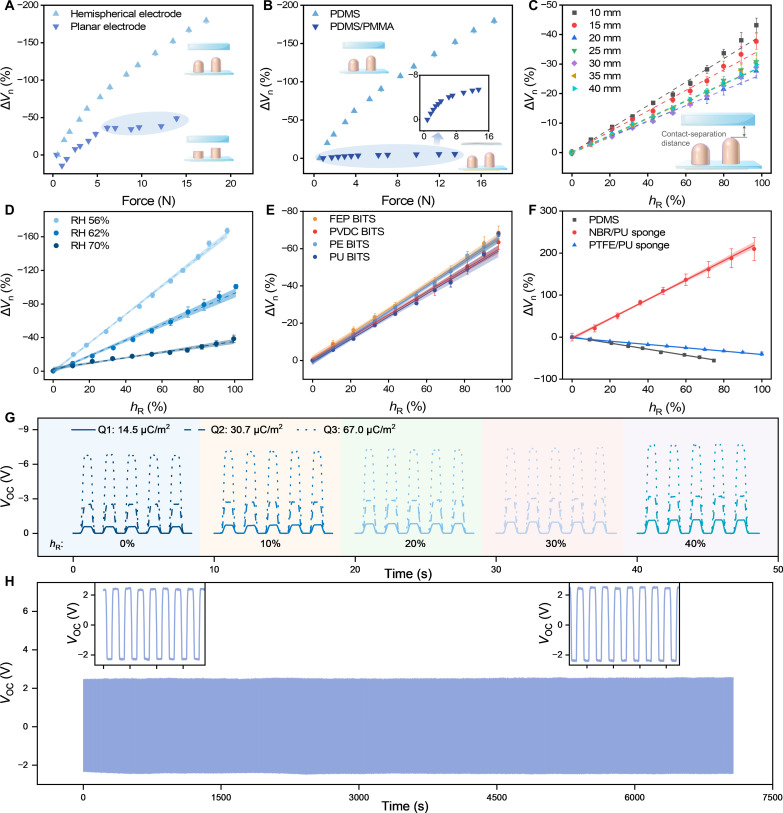
Influencing factors of the BITS units. (**A**) Δ*V*_n_ of the hemispherical and planar heads BITS as force increases. (**B**) Relationship between Δ*V*_n_ and contact force of PDMS elastomer and PDMS/PMMA using the hemispherical head BITS (blue region: constant region). Relationship between Δ*V*_n_ and *h*_R_ at (**C**) varying contact-separation distances and (**D**) different humidity environments. The relationship between Δ*V*_n_ and *h*_R_ for (**E**) BITS units with different triboelectric layers (FEP, PVDC, PE, and PU, respectively) and (**F**) test samples of PDMS elastomer, NBR/PU sponge, and PTFE/PU sponge. (**G**) Voltage curve of different surface charge densities. (**H**) Voltage curves and enlarged figure of the contact cycle test.

In general, the structural design of the BITS and environmental factors exert an influence on TENG-based output performance. Therefore, it is necessary to ensure the stability of the sensing performance of the BITS under different structural designs and environmental conditions. To validate the effectiveness of the hemispherical structure of the BITS, the performance between the hemispherical and planar heads was compared (Fig. 3A and figs. S8A and S9). Because of the increase in the contact area, the magnitude of Δ*V*_n_ from hemispherical heads increased in step gradually from 0 to 180% as the contact force increased from 0 to 16 N. On the contrary, the contact area between planar heads and samples remained unchanged during the contact process when the contact force *F* was beyond a threshold of 5 N, resulting in a stable Δ*V*_n_. Furthermore, COMSOL simulation supported the distinct *V*_OC_ characteristics under different contact heights between the hemispherical and planar electrodes, highlighting the advantages of the hemispherical structure (fig. S8B). As shown in Fig. 3B, hemispherical heads can recognize both soft and hard samples due to different Δ*V*_n_ responses versus the force, in which the Δ*V*_n_ of both PDMS and PDMS/PMMA increased with the force. Consequently, it is essential to use a hemispherical head to detect the objects’ softness accurately.

The contact-separation distance between BITS and the sample can also contribute to the open-circuit voltage. The Δ*V*_n_ magnitude varied for each contact-separation distance from 10 to 40 mm (Fig. 3C). Although the Δ*V*_n_ magnitude differed for each distance, the correlation between Δ*V*_n_ and *h*_R_ maintained linear, demonstrating the robustness of the proposed system toward contact-separation distances. Humidity is another critical factor that affects the open-circuit voltage. To further verify the stability of the BITS, the Δ*V*_n_ magnitude versus *h*_R_ was tested at different relative humidity (RH) environments (Fig. 3D). The Δ*V*_n_ decreased gradually with increasing humidity, which can be attributed to the reduced charge density. However, the relationship between Δ*V*_n_ and *h*_R_ remained linear, making it applicable for contact height detection irrespective of environmental humidity conditions.

The triboelectric charge density is highly related to the electron affinities of triboelectric surfaces. Thus, this approach can be extended to different material pairs, encompassing both triboelectric materials and test samples. To demonstrate this approach, BITS units with various triboelectric layers, including FEP, polyvinylidene chloride (PVDC), polyethylene (PE), and polyurethane (PU), were used to contact PDMS (Fig. 3E). The results suggested that Δ*V*_n_ were associated with the distinct electronegativity of the triboelectric layer, presenting a linear relationship with *h*_R_. On the contrary, the *V*_OC,W_ exhibited a parabolic relationship with *h*_R_ within the effective contact range of the hemispherical head, according to Eq. 3 (fig. S10). Moreover, this strategy was applicable for test samples of different electronegativities, demonstrated with materials including PDMS elastomer, NBR/PU sponge, and polytetrafluoroethylene (PTFE)/PU sponge using the FEP BITS unit, positioned at different sequences in the triboelectric series (Fig. 3F). These results highlighted the universal applicability of this approach across various material combinations and provided strong evidence for using triboelectric signals to determine material softness. Furthermore, the linear relationship between Δ*V*_n_ and *h*_R_ was maintained using BITS units with diameters from 500 μm to 5 mm (fig. S11), demonstrating the potential of miniaturization.

The charge density represents a pivotal parameter influencing output voltage. In this study, we used PDMS samples with varying surface charge densities to evaluate the impact of surface charge density on contact height detection (Fig. 3G and fig. S12). The *V*_OC_ showed an increase as the surface charge density increased, consistent with Eqs. 3 and 4. Furthermore, the validity of this output pattern was further confirmed through FEA using COMSOL (fig. S13). As a result, the BITS is capable of a broad range of surface charge densities. The BITS also demonstrated long-term stability for more than 5000 testing cycles, and the *V*_OC_ maintained a consistent amplitude without any discernible alterations or attenuation, as illustrated in Fig. 3H.

### Softness identification for various materials

**Fig. 4.**
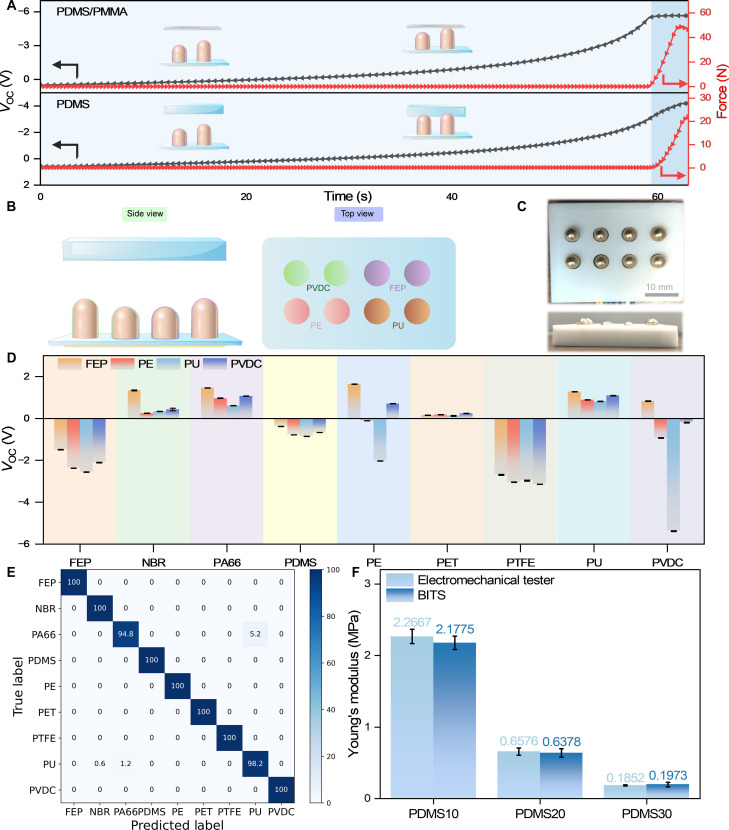
Softness identification and modulus quantification. (**A**) Approaching and touching process using BITS with PDMS elastomer and PDMS/PMMA. (**B**) Illustration and (**C**) photograph of the BITS array. (**D**) Open-circuit voltage of different objects with the BITS array. (**E**) Machine learning confusion matrix for recognizing material type. (**F**) Modulus quantification comparison between the BITS and a universal mechanical test machine.

The capability for softness identification of the BITS was further evaluated by PDMS elastomer and PDMS/PMMA (Fig. 4A). In the initial state, the sample and BITS were positioned at a distance exceeding 50 mm to avoid electrostatic induction. As the BITS and the sample approached, induced charges began to develop, increasing the induced potential. During the approaching process, the sensor generated an identical *V*_OC_ response for both samples before physical contact. When the BITS contacted the sample, *V*_OC_ exhibited notable differences. With a gradual increase in contact force, the *V*_OC_ of PDMS/PMMA remained unchanged, whereas that of PDMS elastomer steadily increased correlated to the increasing contact force, primarily due to variations in the contact area. These results further supported the sensor’s capability to perceive and differentiate materials of varying softness during touch.

Regarding object perception, the material type is a crucial element of haptic sensing. The electron affinity of a material dictates electron gain or loss, with charge transfer magnitude depending on the material’s electronegativity. By evaluating the type and amount of the transferred charges, it became possible to ascertain the position of the test sample in the triboelectric series and subsequently determine the possible material type. The BITS array composed of four triboelectric materials (FEP, PVDC, PE, and PU, respectively, as shown in Fig. 4, B and C) was used to identify a wide range of test materials. Initially, when contacting the PTFE with large electronegativity (fig. S14), the BITS array with four different triboelectric layers generated trough-to-peak signals with different *V*_OC_ values due to the varying electron affinities of materials, leading to different charge transfer amounts. Subsequently, when NBR was used as the test material, the BITS array exhibited distinct waveform signals from a peak to a trough with different *V*_OC_ values. The BITS array was also used to detect various materials (Fig. 4D), with unique waveforms and corresponding output characteristics. These results demonstrated the ability of the BITS array to identify the material types based on triboelectric open-circuit voltage characteristics.

On the basis of the accurate material type and softness recognition capability of the BITS array, machine learning techniques can effectively extract features, conduct data analysis, and make high-accuracy predictions of material types and softness. By using machine learning, the accuracy and efficiency of material identification can be enhanced with the reduced time and computational resources consumed in signal feature extraction. Using the *k*-nearest neighbors (KNN) algorithm, the BITS array accurately identified material types and softness by machine learning. Figure S15 presents a flowchart that described the details of material type and softness identification by the BITS array. Initially, the model was trained using prelabeled data. Triboelectric electrical signals and corresponding pressures were collected using the BITS array to touch different test materials. Nine materials were tested to train the model for further identification. After model training, the trained model was used to recognize material type and softness. As shown in Fig. 4E, the material types can be classified accurately with a high accuracy rate of 99.4% by integrating signals from different electrodes.

In addition, when the BITS contacted the elastomer, the measured contact height increased with the decrease in the compressive modulus of the elastomer, which determined the material softness. Therefore, the BITS array can predict and identify the softness of the materials with an accuracy rate of 100% (fig. S16). By identifying material type and softness, the BITS array can precisely quantify the modulus of different materials. The Young’s modulus of elastomers can be calculated from the Hertz model with the BITS array, as derived from Eq. 1. Through this strategy, we tested PDMS with different softness fabricated by varying cross-linking degrees. The results showed that the BITS can accurately recognize Young’s modulus magnitude, consistent with the results of the universal mechanical testing machine (Fig. 4F and figs. S17 and S18), confirming the accuracy of our BITS in recognizing Young’s modulus. Taking advantage of the BITS system, a wide range of haptic parameters (material softness, Young’s modulus, contact height, and material type) can be detected with high accuracy compared to other technologies (table S1).

### Demonstration of intelligent tactile perception system

**Fig. 5.**
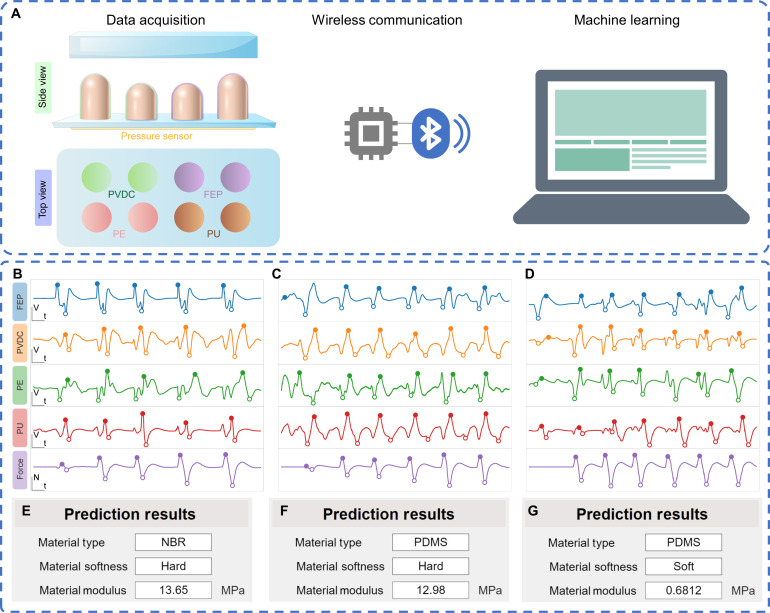
Application of material type and softness recognition achieved by the BITS array. (**A**) Illustration of the structure, sensing process, and machine learning. (**B** to **D**) Output signals of the BITS array and (**E** to **G**) the recognition and prediction results of NBR/PMMA, PDMS/PMMA, and PDMS20, respectively.

To validate the feasibility of BITS in enabling robots to perceive objects in the surrounding environment, we developed an intelligent tactile perception system (ITPS) for tactile perception. This system comprised a BITS array for material type and softness detection, a pressure sensor for pressure measurement, an Microcontroller Unit (MCU)–based data processing and acquisition module, and a data transmission module (Fig. 5A). The BITS array and the pressure sensor captured data of contact height and contact force, respectively, which was further collected using a high-precision multichannel data acquisition device, processed by the MCU, and transmitted to a computer for real-time machine learning and prediction. This analysis enabled the determination of the material’s type and softness. The intelligent softness recognition system applied varying forces during consecutive touches on the test sample to obtain continuously changing signals from the BITS array and the pressure sensor. The MCU collected, processed, and transmitted these data to a computer for machine learning. The prediction results were visually displayed on the computer. The ITPS system collected and displayed waveforms and voltage magnitude signals during the touch of three representative materials with different softness and electron affinities, including NBR/PMMA, PDMS/PMMA, and PDMS20, respectively (Fig. 5, B to D, and fig. S19), and then their material type and softness were successfully predicted. Such the prediction capability was also applied on other materials, including FEP/PMMA, PTFE/PMMA, PVDC/PMMA, PE/PMMA, Polyamide66/PMMA (PA66/PMMA), PU/PMMA, NBR/PMMA, NBR/PU sponge, PDMS, and Ecoflex (Fig. 5, E to G, fig. S20, and movie S1). In addition, by integrating the pressure sensor results, the system can further calculate Young’s modulus.

## DISCUSSION

Inspired by insects’ antennae, we proposed a strategy for tactile perception by identifying the material type and quantifying Young’s modulus of objects using the triboelectric effect. By identifying the contact height, contact area, and pressure magnitude, the BITS can perceive the softness of the object. In addition, due to the varying ability of materials to gain or lose electrons, the BITS array generated unique triboelectric signals when in contact with different objects, thereby determining the potential material type. Building upon these principles, we developed an ITPS to identify various material types and softness with quantified Young’s modulus of the materials. Furthermore, the BITS array can enhance robotic and prosthetic tactile perception systems, empowering them to perceive the softness of objects, which has potential applications in clinical examination, such as detecting tissue swelling, thereby expanding the depth and breadth of remote healthcare. We believe tactile simulation technology holds immense potential in medical rehabilitation and the intelligence industry.

## MATERIALS AND METHODS

### Fabrication of softness haptic perception system

Single-electrode mode TENGs were fabricated using high-purity polymer materials (FEP, PVDC, PE, and PU film) as the triboelectric layer attached on the surface of the hemisphere copper pillars with a diameter of 5 mm, named FEP BITS, PVDC BITS, PE BITS, and PU BITS, respectively. The lower copper pillar with a 1-mm lower height with the triboelectric layer was used as a reference electrode, whereas the upper one was used as the working electrode. Then, each copper pillar with a triboelectric layer was fixed on the printed circuit board (PCB) with 10-mm pad spacing to fabricate a BITS array. The BITS array in microscale was manufactured with a high-resolution 3D printer (nanoArch S140, BMF Precision Tech Inc., China) and patterned Ag as electrodes. The resistive pressure sensor (Flexiforce A201; 0 to 110 N and accuracy of 0.01 N) with a sandwich structure was applied to the backside of the PCB to measure the contact pressure. A multichannel open-source module (OpenBCI) with ADS1299 analog-to-digital converter (ADC) (Texas Instruments; 24-bit channel resolution and 16-kHz sampling rate) was used as the data acquisition module, and the Bluetooth Low Energy module was used as the wireless data communication module between the ADC and the computer.

### Sample preparation

PDMS with different cross-link degrees were synthesized at 60°C by adjusting the precursor and cross-linker weight ratio with 10:1, 20:1, and 30:1, named PDMS10, PDMS20, and PDMS30, respectively. Ecoflex (Smooth-On) rubber samples (00-10, 00-20, and 00-30) were fabricated by mixing corresponding components A and B with a 1:1 weight ratio and cured at room temperature, named Ecoflex 00-10, Ecoflex 00-20, and Ecoflex 00-30, respectively. For PDMS/PMMA, a thin film was attached to a hard PMMA substrate with a size of 50 mm by 50 mm by 2 mm. For NBR/PU sponge, a thin film was attached to a soft PU sponge with a size of 50 mm by 50 mm by 3 mm. Other films on hard PMMA and/or soft PU sponge were made through the same process.

### Characterization and measurement

The BITS array was placed on a force gauge (IMADA ZTS-100 N), and the test sample was fixed on the linear motor (LinMot H01) to contact the BITS array with a displacement range from 0 to 2.5 mm. The displacement of the linear motor and corresponding contact force was recorded through a computer. The stability cycle test was performed using the linear motor with a contact force of 5 N for over 5000 cycles. TENG’s electrical output performance (open-circuit voltage, short-circuit current, and short-circuit charge) was tested and recorded by an electrostatic meter Keithley 6514 (Tektronix). To determine the contact height *h*_R_ from Δ*V*_n_, a linear relationship is established using two test points: the initial point at zero contact height and force to obtain open-circuit voltage *V*_0_ and another point at a specific contact height to get *V_h_* and calculate Δ*V*_n_. This linear relationship allows the calculation of *h*_R_ from the measured Δ*V*_n_, enabling the derivation of Young’s modulus value from Eq. 1 when combined with the corresponding contact force (see abbreviation list in table S2). To adjust the surface charge density of PDMS, a contact-separation mode TENG with the triboelectric layer FEP was used to measure the transferred charge by Keithley 6514 for charge density calculation. Machine learning was achieved using the KNN algorithm by MATLAB on the computer. A mechanical compress test was performed by an electromechanical universal testing machine with a 500-N force sensor (MTS EM Compression).

## Supplementary Materials

### This PDF file includes:

Supplementary Texts S1 and S2 Figs. S1 to S20 Tables S1 and S2 Legend for movie S1 References
