# Supplementary material for: Biomimetic bimodal haptic perception using triboelectric effect
Source: Sci Adv. 2024 Jul 5;10(27):eado6793. doi: 10.1126/sciadv.ado6793 (PMC11225791; doi:10.1126/sciadv.ado6793)
Supplement: Supplementary file 1 — Supplementary Texts S1 and S2 Figs. S1 to S20 Tables S1 and S2 Legend for movie S1 References [file sciadv.ado6793_sm.pdf]

Science Advances
AAAS

Supplementary Materials for

Biomimetic bimodal haptic perception using triboelectric effect

Shaoshuai He *et al.*

Corresponding author: Yunlong Zi, ylzi@hkust-gz.edu.cn; Xin Xia, xinxia@hkust-gz.edu.cn; Xiya Yang, xiyayang@jnu.edu.cn

Sci. Adv. **10**, eado6793 (2024)
DOI: 10.1126/sciadv.ado6793

The PDF file includes:

Supplementary Texts S1 and S2
Figs. S1 to S20
Tables S1 and S2
Legend for movie S1
References

Other Supplementary Material for this manuscript includes the following:

Movie S1

Supplementary Text

Supplementary Text S1: Relationship between Young's modulus and contact height.

Hertz found that for the contact between a spherical indenter and a planar sample (Fig. S3), the radius of the maximum contact area *r* is related to the contact force *F* and the mechanical properties of materials, given by (*16*, *17*):

$$r^3 = \frac{3}{4}\frac{FR}{E^*} \qquad \text{(Equation S1)}$$

Where *R* is the radius of the indenter, E^* is the effective Young's modulus consisting of the Young's modulus of the indenter E_i and the sample E_s:

$$\frac{1}{E^*} = \frac{1-v_i^2}{E_i} + \frac{1-v_s^2}{E_s} \qquad \text{(Equation S2)}$$

Where v_i and v_s are the Poisson's ratio of the indenter and the sample, respectively, depending on the material's intrinsic property. For a rigid indenter and a soft material contact, considering $E_i \gg E_s$, Equation S2 can be simplified and rearranged as follows:

$$E^* = \frac{E_s}{1-v_s^2} \qquad \text{(Equation S3)}$$

The contact area between the spherical indenter and the soft sample can be regarded as a sphere crown. The radius of the circle of the largest open part of the sphere crown is *r*, and the height of the sphere crown is *h* (Fig. S3). Therefore, the contact area *S* can be obtained by:

$$S = 2\pi Rh \qquad \text{(Equation S4)}$$

r can also be given by:

$$r^2 = R^2 - (R-h)^2 = 2Rh - h^2 \qquad \text{(Equation S5)}$$

Combining Equations S1, S3, and S5, the sample modulus (E_s) can be derived from

$$E_s = \frac{3}{4}FR\left(1-v_s^2\right)\left(2Rh-h^2\right)^{-\frac{3}{2}} \qquad \text{(Equation S6)}$$

Poisson's ratio is a material property and has been recognized as a function of the typical atomic packing density (*36*). The Poisson's ratio of each material can be determined through experiments:

$$\nu = -\frac{\varepsilon_y}{\varepsilon_x} \qquad \text{(Equation S7)}$$

Where ε_y is deformation along the force direction, ε_x is the deformation perpendicular to the force direction. Furthermore, the Poisson's ratio can be modeled from empirical data related to the molecular cross-sectional area A (*37*):

$$\nu = 0.513 - 2.37 \times 10^6 \sqrt{A} \qquad \text{(Equation S8)}$$

The molecular cross-sectional area A is related to the van der Waals volume V_w and the length of the repeat unit in its fully extended conformation l_m:

$$A = \frac{V_w}{N_A l_m} \qquad \text{(Equation S9)}$$

N_A is Avogadro's number. Therefore, the Poisson's ratio can be determined based on the material type.

Supplementary Text S2: Relationship between TENG open-circuit voltage and contact height.

According to the theoretical model for a contact-mode TENG, the open-circuit voltage V_{OC} can be described as:

$$V_{OC} = \frac{\sigma S}{C} \quad \text{(Equation S10)}$$

Where σ is the charge density, S is the effective area of induced potential. C is the electric capacitance of the TENG.

To determine the relationship between the open-circuit voltage V_{OC} and contact height h during the contact process, it is assumed that the contact-separation distance between the sample and the electrode is infinitely small, thereby the V_{OC} during the approaching process can be ignored. Before contact, the elastomer is flat, and the effective area can be given by the projection of the contact area (hemispherical crown) of the indenter:

$$S = \pi r^2 = \pi \left(2Rh - h^2\right) \quad \text{(Equation S11)}$$

After contact, the corresponding area increases caused by deformation. The effective contact area is the spherical crown area according to Equation S4:

$$S' = 2\pi Rh \quad \text{(Equation S12)}$$

The transferred short-circuit charge Q_{SC} during the contact process is considered proportional to the area change:

$$Q_{SC} = \sigma \left(S' - S\right) = \sigma \pi h^2 \quad \text{(Equation S13)}$$

Therefore, the open-circuit voltage can be obtained by:

$$V_{OC} = \frac{\sigma \pi h^2}{C} \quad \text{(Equation S14)}$$

However, the capacitance may be affected by the relative displacement from untouched to contact process, inducing a small change in voltage δV, therefore the open-circuit voltage can be given as Equation 3 in the manuscript:

$$V_{OC} = \frac{\sigma \pi h^2}{C} + \delta V \quad \text{(Equation S15)}$$

When there is a reference electrode with a height lower Δh, the open-circuit voltage can be regarded as the voltage difference $V_{OC,r}$ between the working $V_{OC,W}$ and reference $V_{OC,RE}$ electrodes, which can be calculated as Equation 4 in the manuscript (assumption that the electric capacitance of the two electrodes is the same):

$$V_{\mathrm{OC}} = V_{\mathrm{OC,W}} - V_{\mathrm{OC,RE}} = \left(\frac{\sigma\pi h^2}{C} + \delta V - \frac{\sigma\pi \left(h - \Delta h\right)^2}{C} - \delta V \right) = \frac{\sigma\pi}{C}\left(2h\Delta h - \Delta h^2\right) \quad \text{(Equation S16)}$$

Considering the contact-separation distance, the open-circuit voltage during the approaching process can be obtained:

$$V_{\mathrm{OC}} = \frac{\sigma\pi R^2}{C_a} \quad \text{(Equation S17)}$$

Where C_a is the capacitance of TENG during the approaching process. Therefore, the open-circuit voltage whole process including approaching and contact can be calculated as Equation 5 in the manuscript considering the contact-separation distance:

$$V_{\mathrm{OC}} = \frac{\sigma\pi R^2}{C_a} + \frac{\sigma\pi}{C}\left(2h\Delta h - \Delta h^2\right) \quad \text{(Equation S18)}$$

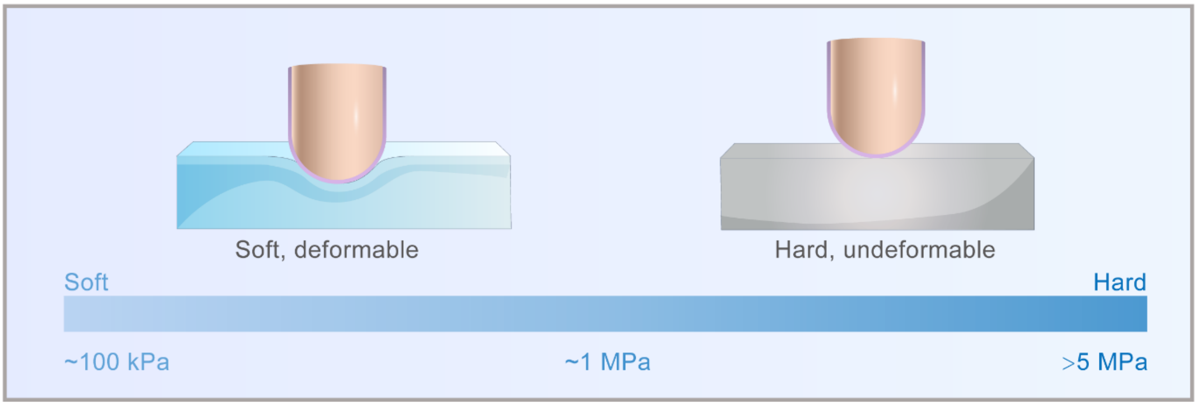

Fig. S1. The material softness reflected by deformability related to Young's modulus.

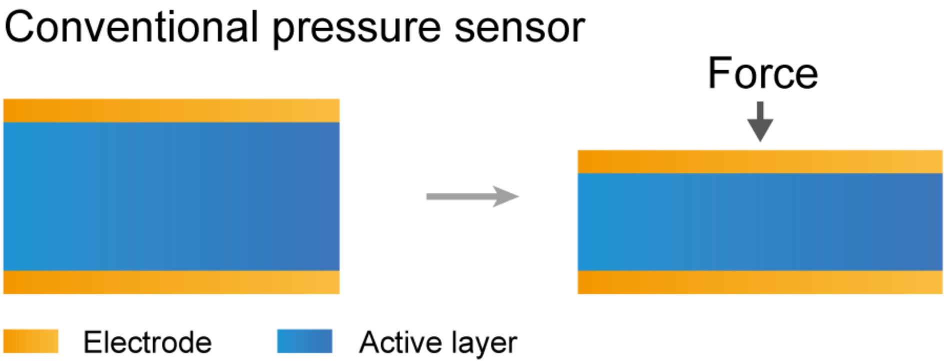

Fig. S2. Working mechanism of conventional pressure sensors.

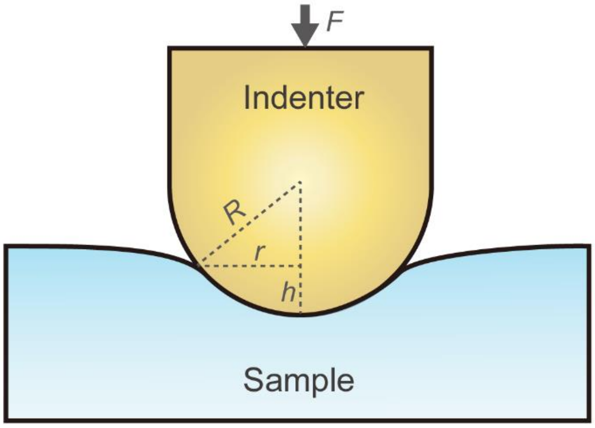

Fig. S3. The Hertz model illustration between a hemispherical indenter and a planar sample.

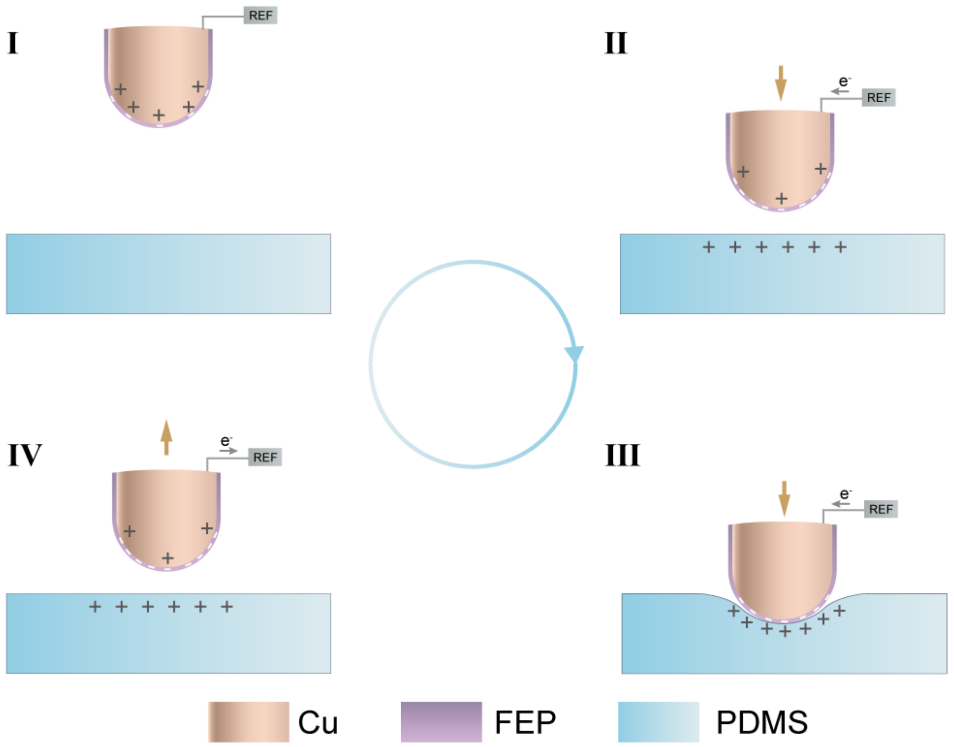

Fig. S4. The working mechanism of triboelectric sensing based on a single-electrode contact-separation mode TENG.

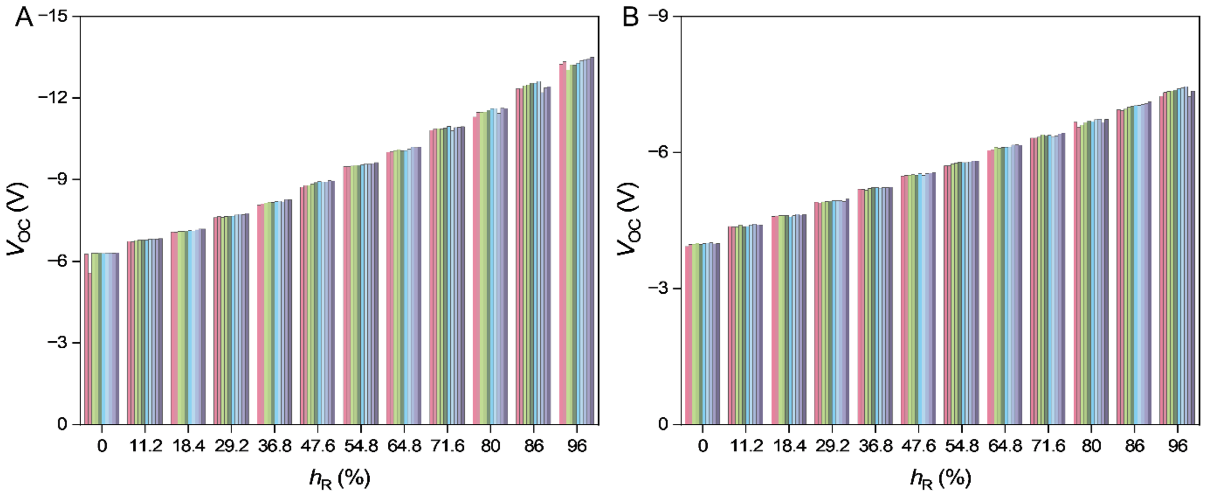

Fig. S5. The open-circuit voltage V_{OC} between PDMS and FEP BITS under each contact height h_R for ten cycles (A) without and (B) with a reference electrode, respectively.

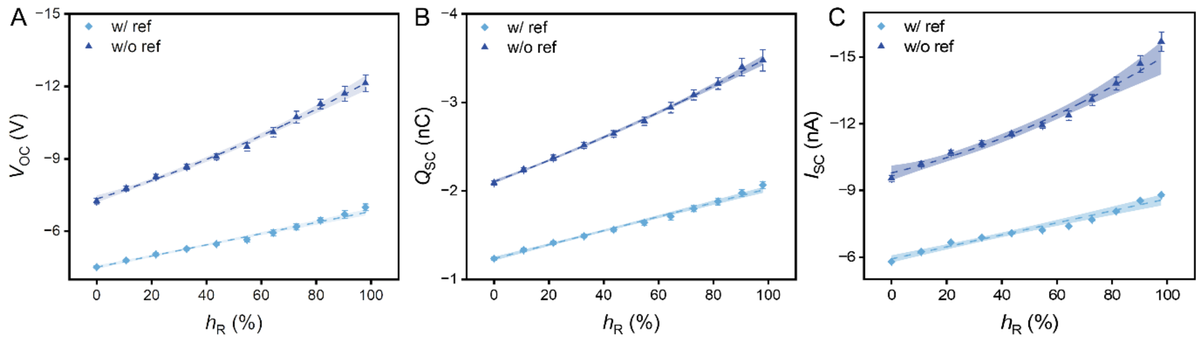

Fig. S6. The BITS output characteristics versus contact height h_R. The relationship between (A) open-circuit voltage, (B) transferred short-circuit charge, (C) short-circuit current and contact height h_R.

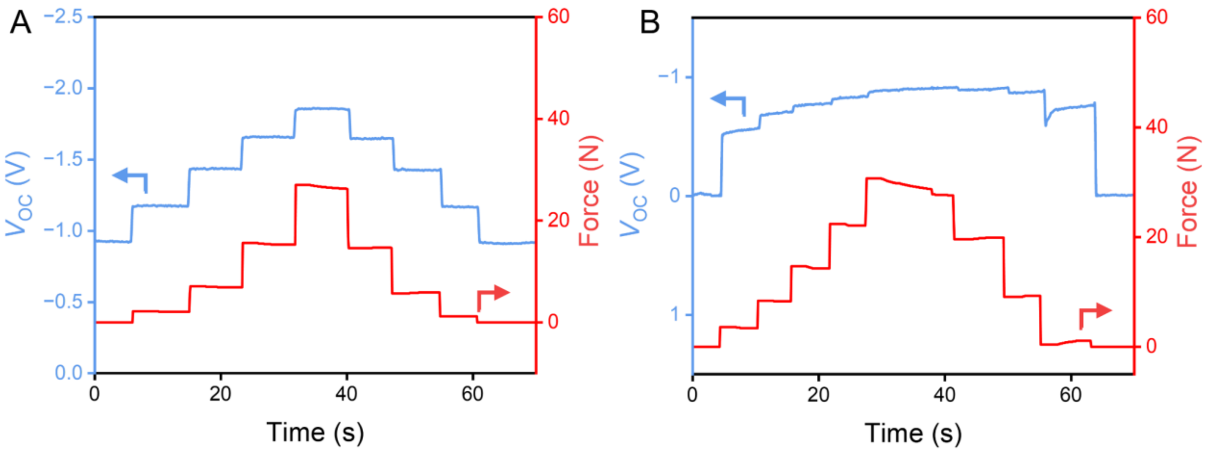

Fig. S7. Continuous open-circuit voltage curves when pressing (A) PDMS and (B) PDMS/PMMA using a FEP BITS.

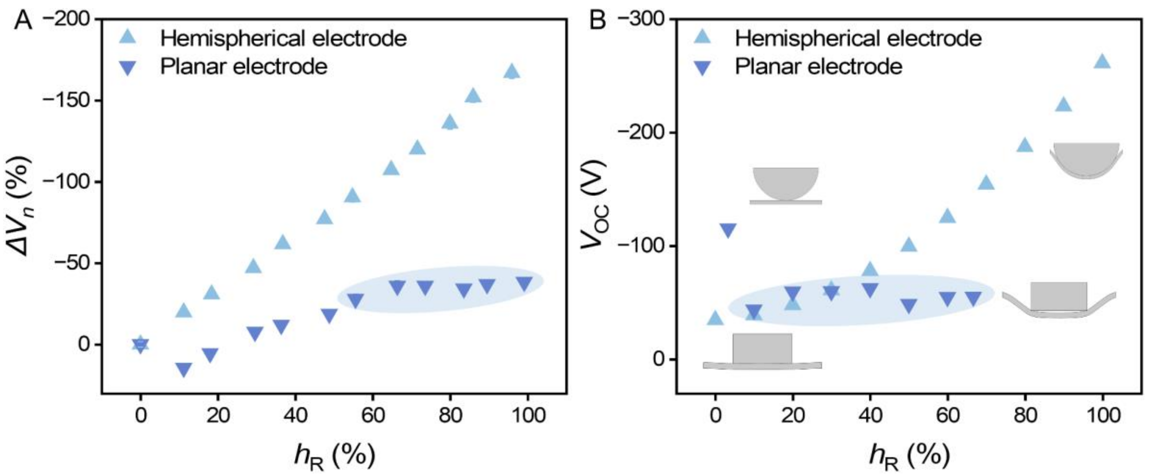

Fig. S8. The comparison between hemispherical and planar electrodes. (A) The relationship between ΔV_n and normalized contact height h_R of hemispherical and planar electrodes. (B) COMSOL simulation results of V_{OC} under varying h_R of hemispherical and planar electrodes (Blue regions: constant region).

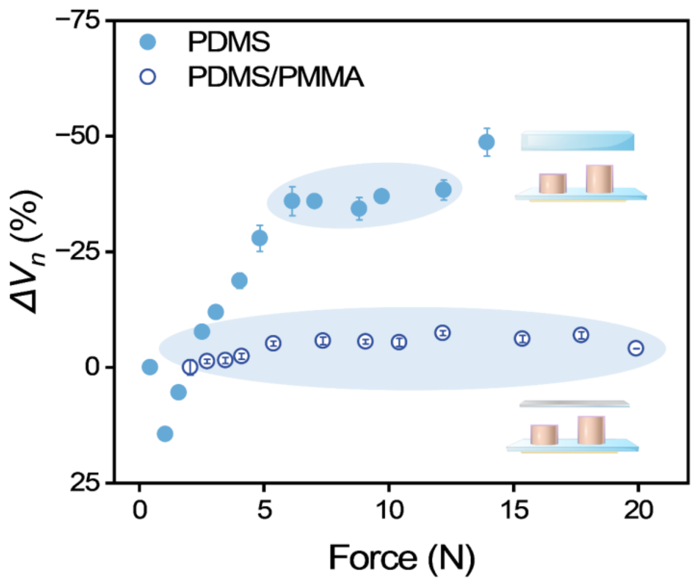

Fig. S9. Relationship between ΔV_n and contact force of PDMS and PDMS/PMMA using the planar head BITS (Blue regions: constant region).

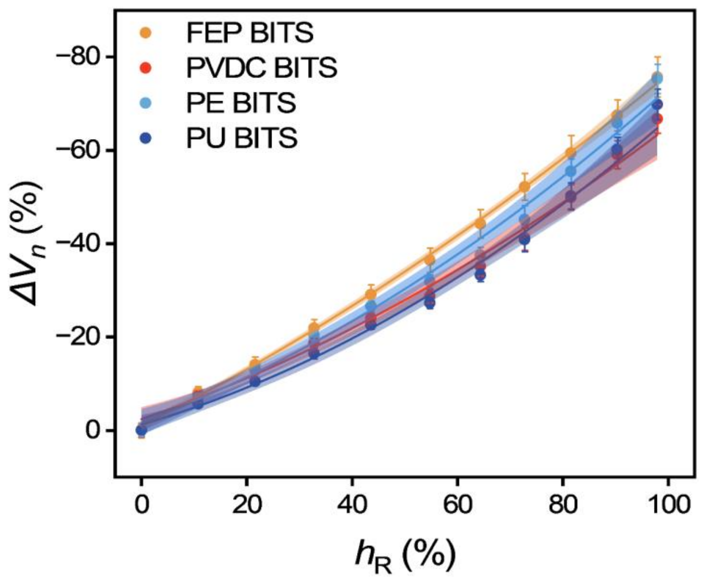

Fig. S10. The ΔV_n of hemispherical head BITS units with different triboelectric layers without a reference electrode.

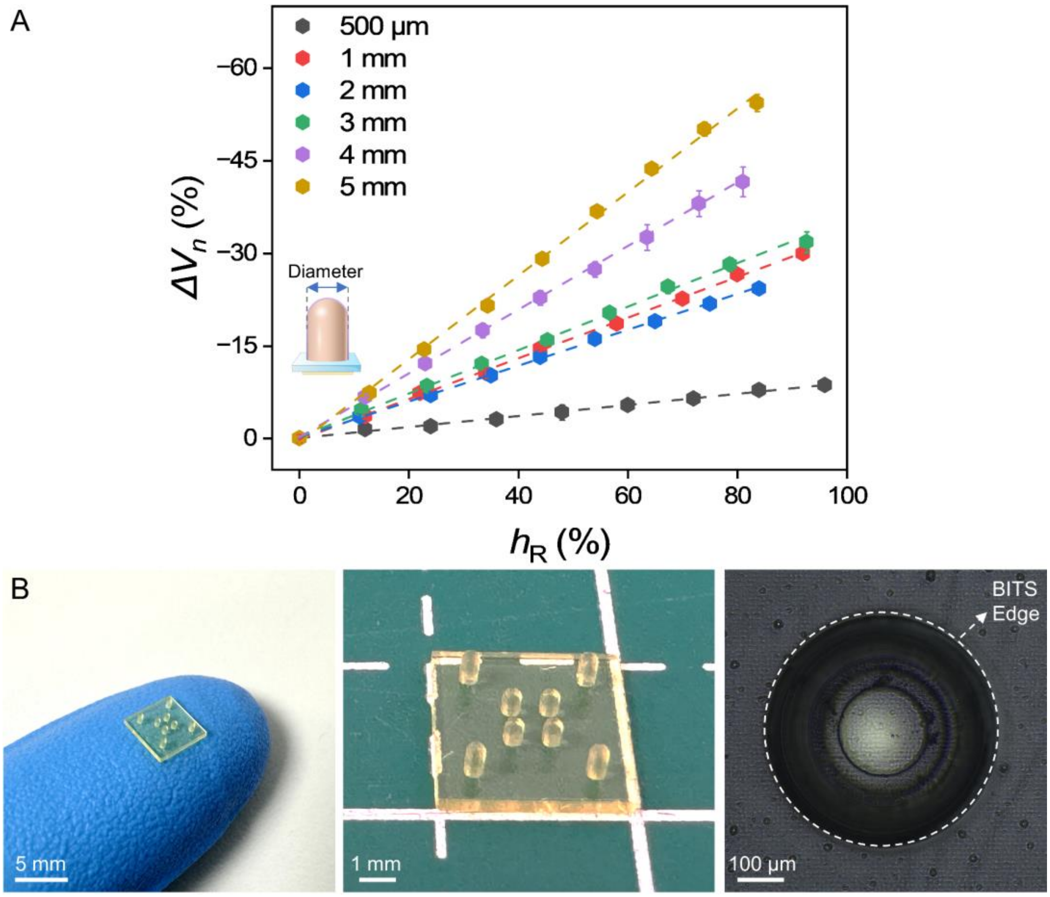

Fig. S11. BITS units with different diameters. (A) The relationship between ΔV_n and h_R of BITS with diameters from 500 μm to 5 mm. (B) Photographs of BITS with microstructures (diameter: 500 μm).

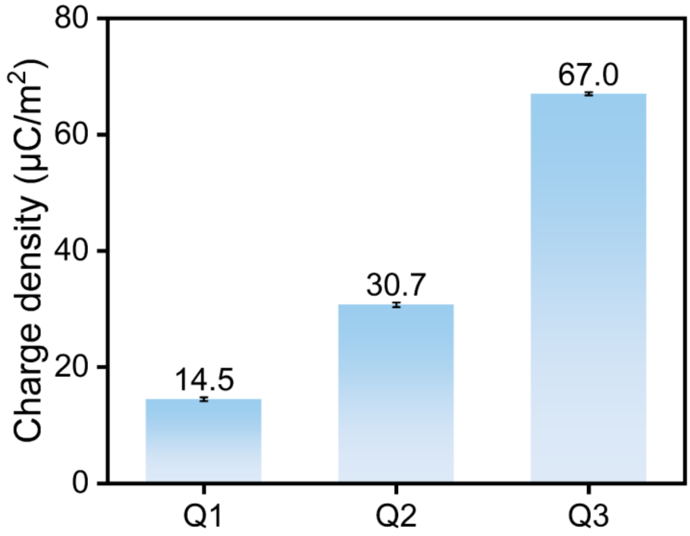

Fig. S12. The surface charge density of each sample.

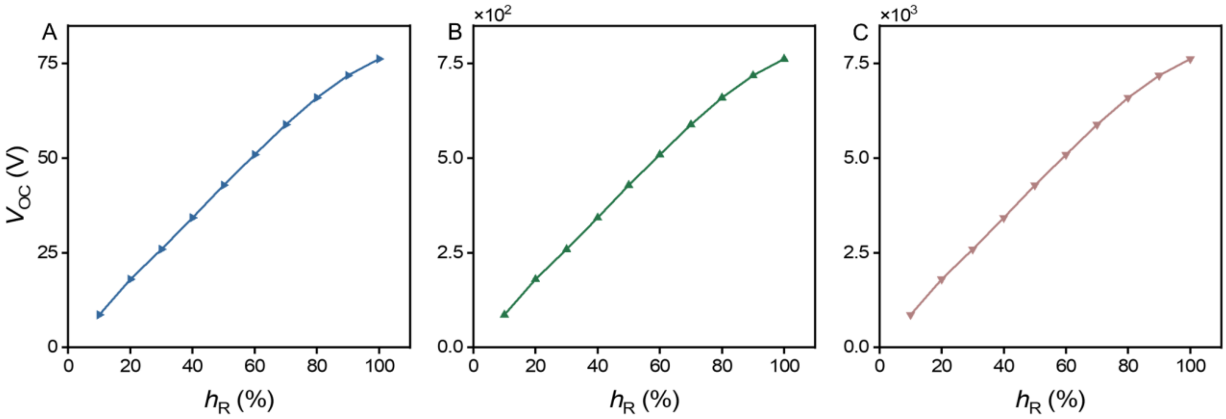

Fig. S13. The voltage changes at different surface charge densities by COMSOL Multiphysics simulation.

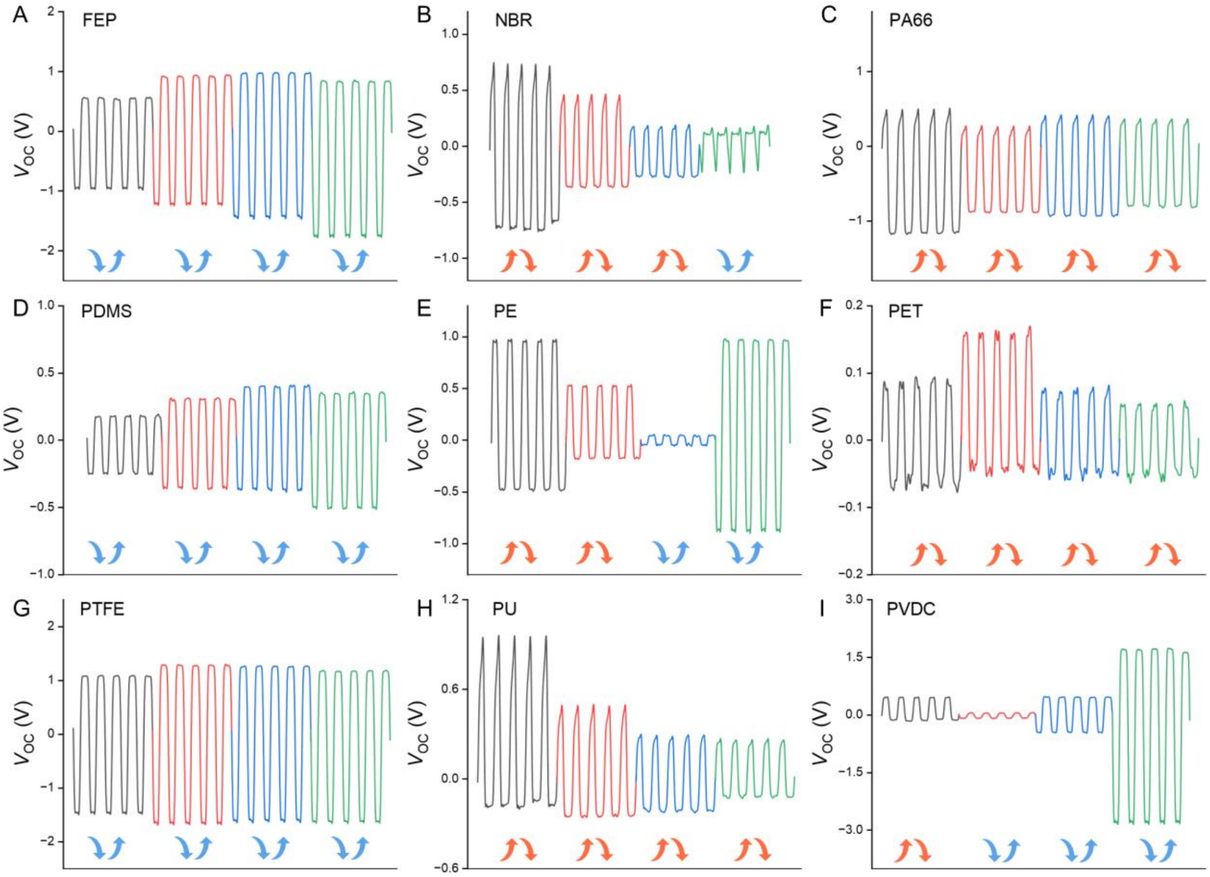

Fig. S14. The open-circuit voltage feature for detecting material type using a BITS array.

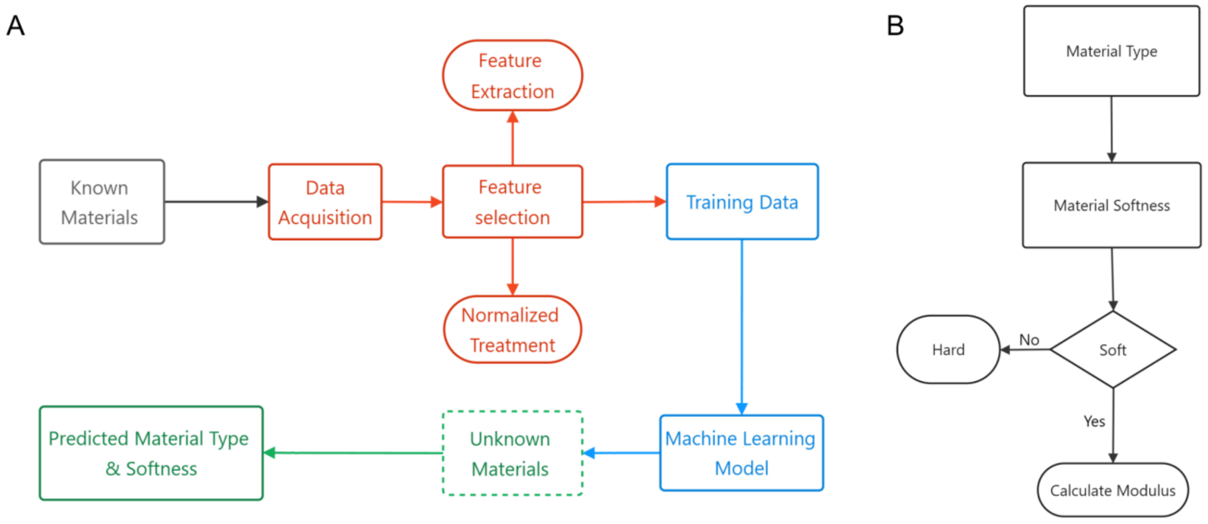

Fig. S15. Machine learning flowchart of (A) material type and softness identification and (B) modulus quantification.

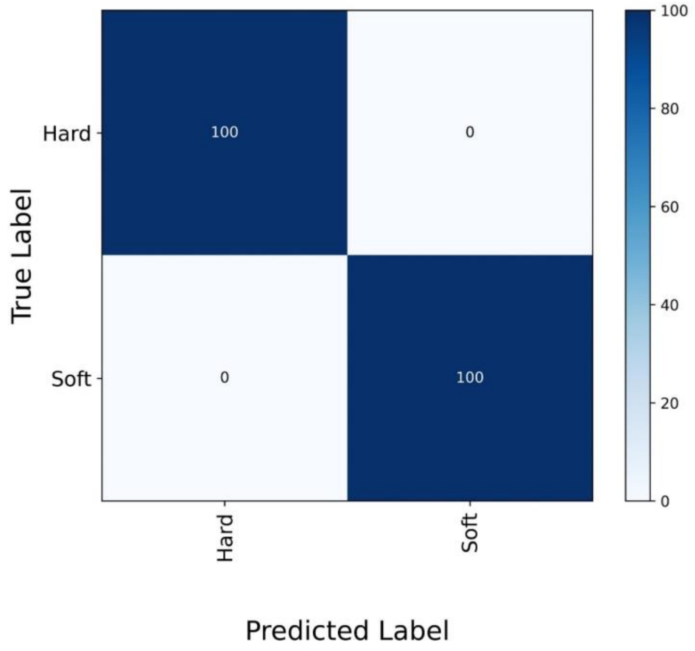

Fig. S16. The machine learning confusion matrix for recognizing material softness.

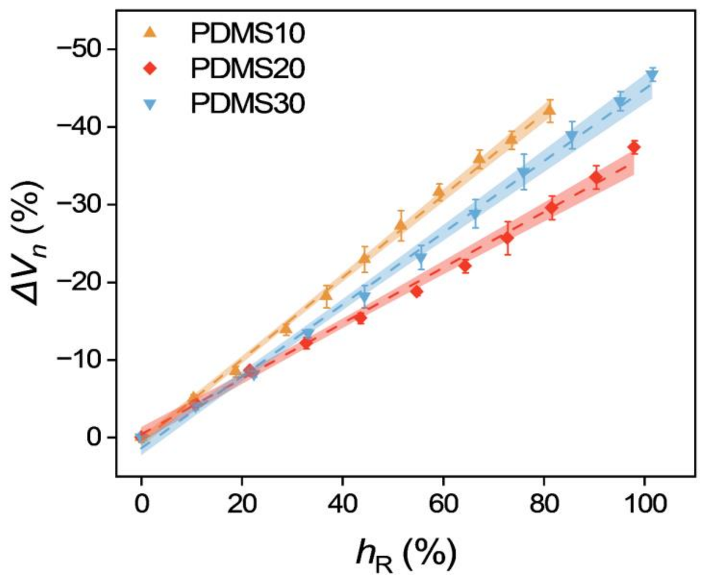

Fig. S17. The ΔV_n as h_R for PDMS samples with different crosslinking degrees.

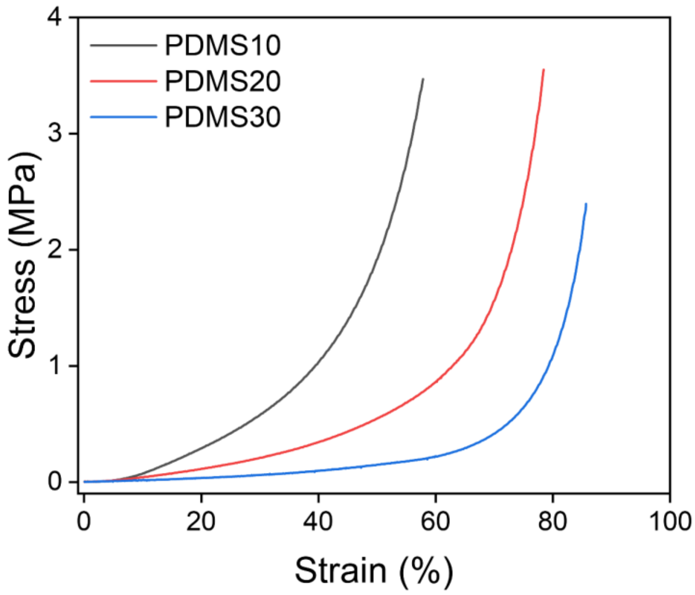

Fig. S18. The compression test of PDMS samples with different crosslinking degrees by a universal mechanical test machine.

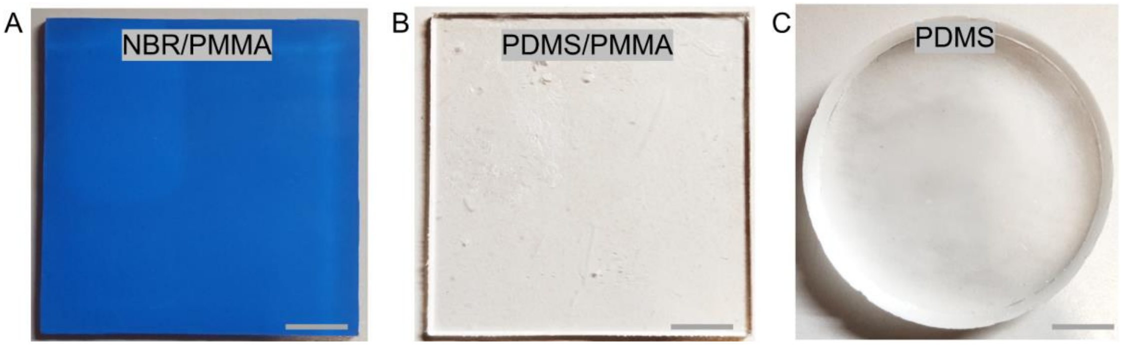

Fig. S19. Photographs for three representative objects of (A) NBR/PMMA, (B) PDMS/PMMA, and (C) PDMS, respectively (scale bar: 10 mm).

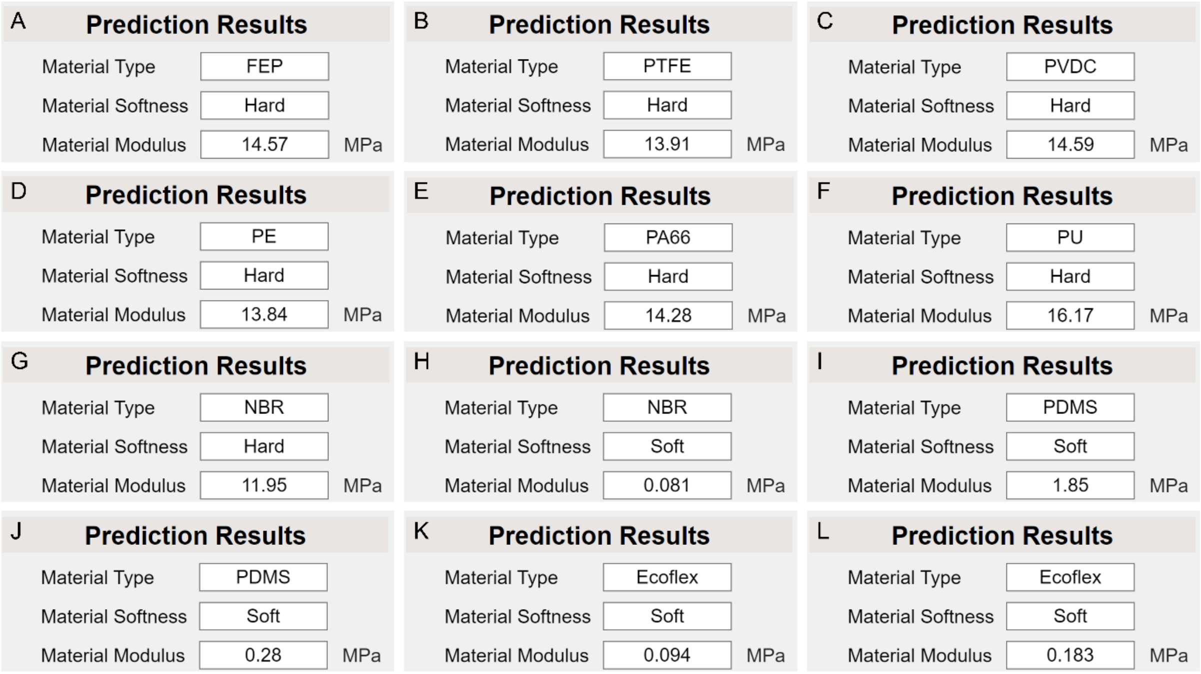

Fig. S20. The recognition and prediction results of a wide range of materials including (A) FEP/PMMA, (B) PTFE/PMMA, (C) PVDC/PMMA, (D) PE/PMMA, (E) PA66/PMMA, (F) PU/PMMA, (G) NBR/PMMA, (H) NBR/PU Sponge, (I) PDMS10, (J) PDMS30, (K) Ecoflex 00-20, and (L) Ecoflex 00-30.

Table S1.

Comparison with other material type and softness identification technologies.

Working mechanism	Device structure	Haptic mode				Force/pressure sensing	Measurement range	AI processing		Ref.
		Material softness	Modulus quantification	Indentation depth/area	Material type			Method	Accuracy	
Triboelectric	**Hemispherical**	√	√	√	√	√	**Samples: PTFE, FEP, PE, PET, PVDC, PU, PA66, NBR, PDMS, Ecoflex**<br>**Young's modulus: 100 kPa to ~ 5 MPa**<br>**Pressure: 0 – 100 N**<br>**Contact-separation distance: 10 – 50 mm**	**KNN algorithm**	**99.4% (type)**<br>**100% (softness)**	**This work**
	Planar	×	×	×	√	×	Test object: PTFE, PVC, silicon, wood, glass, PU, etc.	Linear discriminant analysis (LDA) algorithm	96.80%	(*14*)
	Planar	×	×	×	√	×	Test object: Cloth, wood, paper, glass, acrylic	Convolutional neural network (CNN) model	96.62%	(*15*)
	Sponge	×	×	×	√	×	Test object: Al, paper, ABS, PI, PTFE	Regression model	93.30%	(23)
Resistance	Self-locked structure	√	√	×	×	√	PDMS and Ecoflex, modulus from 37 kPa to 1 MPa	N.A.	N.A.	(*20*)
	Protrusion	√	×	×	×	√	PDMS, modulus from 37 kPa to 1046 kPa	Supporting vector machine for body feature	98%	(*21*)
	Bilayer	√	×	√	×	√	Ecoflex and silicone, with varying cross-link degrees	Supporting vector machine using softness	55%	(*22*)
Resistance and capacitance	Bilayer	√	×	×	×	√	PDMS	N.A.	N.A.	(*38*)
Capacitance	Bilayer	√	√	√	×	√	PDMS	N.A.	N.A.	(*39*)
Computer vision	N.A.	√	×	×	√	√	Artificial plants	Volumetric stiffness field (VSF) model	N.A.	(*40*)
	Gelsight	√	×	√	×	×	Shore 00 17 to 70	CNN and LSTM	N.A.	(41)

Table S2.

Abbreviation list in the article and supporting information.

Abbreviation	Meaning
E^*	Effective modulus of indenter and sample couples
E_s	Compressive modulus of the sample
E_i	Compressive modulus of the indenter
F	Contact force
R	Indenter radius
v_s	Poisson's ratio of the material
v_i	Poisson's ratio of the indenter
S	Contact area
h	Contact height between the BITS and samples
h_R	Normalized contact height, defined as h/R
Δh	Height difference between working and reference electrodes
V_{OC}	Open-circuit voltage
δV	Open-circuit voltage variations caused by capacitance
$V_{OC,W}$	Open-circuit voltage of the working electrode
$V_{OC,RE}$	Open-circuit voltage of the reference electrode
$V_{OC,r}$	Relative open-circuit voltage between working and reference electrodes
V_h	$V_{OC,r}$ at specific contact height
V_0	$V_{OC,r}$ when contact height is zero
ΔV	Absolute open-circuit voltage change, defined as V_h-V_0
ΔV_n	Normalized open-circuit voltage change, defined as $\Delta V/V_{OC,r}$
Q_{SC}	Short-circuit charge
I_{SC}	Short-circuit current
C	Capacitance of the BITS during the contact process
C_a	Capacitance of the BITS during the approaching process
σ	Surface charge density of triboelectric layer

Movie S1.

Demonstration for the ITPS system using BITS array in recognition of material type and softness and quantify the modulus.

REFERENCES AND NOTES

1. Q. Su, Q. Zou, Y. Li, Y. Chen, S. Y. Teng, J. T. Kelleher, R. Nith, P. Cheng, N. Li, W. Liu, S. Dai, Y. Liu, A. Mazursky, J. Xu, L. Jin, P. Lopes, S. Wang, A stretchable and strain-unperturbed pressure sensor for motion interference–free tactile monitoring on skins. *Sci. Adv.* **7**, eabi4563 (2021).

2. S. He, B. Guo, X. Sun, M. Shi, H. Zhang, F. Yao, H. Sun, J. Li, Bio-inspired instant underwater adhesive hydrogel sensors. *ACS Appl. Mater. Interfaces* **14**, 45869–45879 (2022).

3. E. Song, M. Chen, Z. Chen, Y. Zhou, W. Zhou, H. T. Sun, X. Yang, J. Gan, S. Ye, Q. Zhang, Mn^{2+}-activated dual-wavelength emitting materials toward wearable optical fibre temperature sensor. *Nat. Commun.* **13**, 2166 (2022).

4. D. Lei, Q. Zhang, N. Liu, T. Su, L. Wang, Z. Ren, Z. Zhang, J. Su, Y. Gao, Self-powered graphene oxide humidity sensor based on potentiometric humidity transduction mechanism. *Adv. Funct. Mater.* **32**, 2107330 (2021).

5. Y. Pang, X. Xu, S. Chen, Y. Fang, X. Shi, Y. Deng, Z. L. Wang, C. Cao, Skin-inspired textile-based tactile sensors enable multifunctional sensing of wearables and soft robots. *Nano Energy* **96**, 107137 (2022).

6. M. Zarei, G. Lee, S. G. Lee, K. Cho, Advances in biodegradable electronic skin: Material progress and recent applications in sensing, robotics, and human-machine interfaces. *Adv. Mater.* **35**, e2203193 (2023).

7. S. Duan, H. Yang, J. Hong, Y. Li, Y. Lin, D. Zhu, W. Lei, J. Wu, A skin-beyond tactile sensor as interfaces between the prosthetics and biological systems. *Nano Energy* **102**, 107665 (2022).

8. L. Ye, F. Wu, R. Xu, Z. Di, J. Lu, C. Wang, A. Dong, S. Xu, L. Xue, Z. Fan, L. Xu, K. Li, D. Li, A. Kursumovic, R. Zhao, R. Tang, L. Qiu, H. Wang, J. L. MacManus-Driscoll, Q. Jing, W. Li, H. Yang, Face mask integrated with flexible and wearable manganite oxide respiration sensor. *Nano Energy* **112**, 108460 (2023).

9. Z. H. Guo, H. L. Wang, J. Shao, Y. Shao, L. Jia, L. Li, X. Pu, Z. L. Wang, Bioinspired soft electroreceptors for artificial precontact somatosensation. *Sci. Adv.* **8**, eabo5201 (2022).

10. W. Liu, Y. Duo, J. Liu, F. Yuan, L. Li, L. Li, G. Wang, B. Chen, S. Wang, H. Yang, Y. Liu, Y. Mo, Y. Wang, B. Fang, F. Sun, X. Ding, C. Zhang, L. Wen, Touchless interactive teaching of soft robots through flexible bimodal sensory interfaces. *Nat. Commun.* **13**, 5030 (2022).

11. U. Khan, T.-H. Kim, M. A. Khan, J. Kim, C. Falconi, S. W. Kim, Zero-writing-power tribotronic MoS2 touch memory. *Nano Energy* **75**, 104936 (2020).

12. H. J. Yoon, D. M. Lee, Y. J. Kim, S. Jeon, J. H. Jung, S. S. Kwak, J. Kim, S. Kim, Y. Kim, S. W. Kim, Mechanoreceptor-inspired dynamic mechanical stimuli perception based on switchable ionic polarization. *Adv. Funct. Mater.* **31**, 2100649 (2021).

13. D. Kim, Z. Yang, J. Cho, D. Park, D. H. Kim, J. Lee, S. Ryu, S.-W. Kim, M. Kim, High-performance piezoelectric yarns for artificial intelligence-enabled wearable sensing and classification. *EcoMat* **5**, e12384 (2023).

14. X. Qu, Z. Liu, P. Tan, C. Wang, Y. Liu, H. Feng, D. Luo, Z. Li, Z. L. Wang, Artificial tactile perception smart finger for material identification based on triboelectric sensing. *Sci. Adv.* **8**, eabq2521 (2022).

15. X. Wei, B. Wang, Z. Wu, Z. L. Wang, An open-environment tactile sensing system: Toward simple and efficient material identification. *Adv. Mater.* **34**, e2203073 (2022).

16. Z. Guo, M. Hao, L. Jiang, D. Li, Y. Chen, L. Dong, A modified Hertz model for finite spherical indentation inspired by numerical simulations. *Eur. J. Mech. A Solids* **83**, 104042 (2020).

17. S. V. Kontomaris, A. Malamou, Hertz model or Oliver & Pharr analysis? Tutorial regarding AFM nanoindentation experiments on biological samples. *Mater. Res. Express* **7**, 033001 (2020).

18. K. K. Liu, K. T. Wan, Multi-scale mechanical characterization of a freestanding polymer film using indentation. *Int. J. Mater. Res.* **99**, 862–864 (2008).

19. F. Robert, Prediction of contact length, contact pressure and indentation depth of Au/carbon nanotube composite micro electrical contact using finite element modeling. *Appl. Surf. Sci.* **489**, 470–476 (2019).

20. Z. Cui, W. Wang, L. Guo, Z. Liu, P. Cai, Y. Cui, T. Wang, C. Wang, M. Zhu, Y. Zhou, W. Liu, Y. Zheng, G. Deng, C. Xu, X. Chen, Haptically quantifying Young's modulus of soft materials using a self-locked stretchable strain sensor. *Adv. Mater.* **34**, e2104078 (2022).

21. Z. Cui, W. Wang, H. Xia, C. Wang, J. Tu, S. Ji, J. M. R. Tan, Z. Liu, F. Zhang, W. Li, Z. Lv, Z. Li, W. Guo, N. Y. Koh, K. B. Ng, X. Feng, Y. Zheng, X. Chen, Freestanding and scalable force-softness bimodal sensor arrays for haptic body-feature identification. *Adv. Mater.* **34**, e2207016 (2022).

22. W. Lin, Z. Wang, Y. Xu, Z. Hu, W. Zhao, Z. Zhu, Z. Sun, G. Wang, Z. Peng, Self-adaptive perception of object's deformability with multiple deformation attributes utilizing biomimetic mechanoreceptors. *Adv. Mater.* **36**, 2305032 (2024).

23. D. Zhu, J. Lu, M. Zheng, D. Wang, J. Wang, Y. Liu, X. Wang, M. Zhang, Self-powered bionic antenna based on triboelectric nanogenerator for micro-robotic tactile sensing. *Nano Energy* **114**, 108644 (2023).

24. C. A. Aubin, B. Gorissen, E. Milana, P. R. Buskohl, N. Lazarus, G. A. Slipher, C. Keplinger, J. Bongard, F. Iida, J. A. Lewis, R. F. Shepherd, Towards enduring autonomous robots via embodied energy. *Nature* **602**, 393–402 (2022).

25. X. Ji, X. Liu, V. Cacucciolo, M. Imboden, Y. Civet, A. El Haitami, S. Cantin, Y. Perriard, H. Shea, An autonomous untethered fast soft robotic insect driven by low-voltage dielectric elastomer actuators. *Sci. Robot.* **4**, eaaz6451 (2019).

26. C. A. Aubin, R. H. Heisser, O. Peretz, J. Timko, J. Lo, E. F. Helbling, S. Sobhani, A. D. Gat, R. F. Shepherd, Powerful, soft combustion actuators for insect-scale robots. *Science* **381**, 1212–1217 (2023).

27. G. de Croon, J. J. G. Dupeyroux, S. B. Fuller, J. A. R. Marshall, Insect-inspired AI for autonomous robots. *Sci. Robot.* **7**, eabl6334 (2022).

28. A. Skordos, P. H. Chan, J. F. V. Vincent, G. Jeronimidis, A novel strain sensor based on the campaniform sensillum of insects. *Philos. Trans. R. Soc. A* **360**, 239–253 (2002).

29. N. S. Szczecinski, C. J. Dallmann, R. D. Quinn, S. N. Zill, A computational model of insect campaniform sensilla predicts encoding of forces during walking. *Bioinspir. Biomim.* **16**, 065001 (2021).

30. J. F. V. Vincent, S. E. Clift, C. Menon, Biomimetics of campaniform sensilla: Measuring strain from the deformation of holes. *J. Bionic Eng.* **4**, 63–76 (2007).

31. H. Zou, Y. Zhang, L. Guo, P. Wang, X. He, G. Dai, H. Zheng, C. Chen, A. C. Wang, C. Xu, Z. L. Wang, Quantifying the triboelectric series. *Nat. Commun.* **10**, 1427 (2019).

32. C. Dhong, R. Miller, N. B. Root, S. Gupta, L. V. Kayser, C. W. Carpenter, K. J. Loh, V. S. Ramachandran, D. J. Lipomi, Role of indentation depth and contact area on human perception of softness for haptic interfaces. *Sci. Adv.* **5**, eaaw8845 (2019).

33. X. Xia, Z. Zhou, Y. Shang, Y. Yang, Y. Zi, Metallic glass-based triboelectric nanogenerators. *Nat. Commun.* **14**, 1023 (2023).

34. W. Yang, X. Wang, H. Li, J. Wu, Y. Hu, Z. Li, H. Liu, Fundamental research on the effective contact area of micro-/nano-textured surface in triboelectric nanogenerator. *Nano Energy* **57**, 41–47 (2019).

35. Y. Zheng, T. Lin, N. Zhao, C. Huang, W. Chen, G. Xue, Y. Wang, C. Teng, X. Wang, D. Zhou, Highly sensitive electronic skin with a linear response based on the strategy of controlling the contact area. *Nano Energy* **85**, 106013 (2021).

36. G. N. Greaves, A. L. Greer, R. S. Lakes, T. Rouxel, Poisson's ratio and modern materials. *Nat. Mater.* **10**, 823–837 (2011).

37. J. T. Seitz, The estimation of mechanical properties of polymers from molecular structure. *J. Appl. Polym. Sci.* **49**, 1331–1351 (1993).

38. L. Beker, N. Matsuhisa, I. You, S. R. A. Ruth, S. Niu, A. Foudeh, J. B. Tok, X. Chen, Z. Bao, A bioinspired stretchable membrane-based compliance sensor. *Proc. Natl. Acad. Sci. U.S.A.* **117**, 11314–11320 (2020).

39. Q. Zou, F. Yang, Y. Wang, Highly sensitive flexible modulus sensor for softness perception and clinical application. *J. Micromech. Microeng.* **32**, 035004 (2022).

40. S. Yao, K. Hauser, Estimating tactile models of heterogeneous deformable objects in real time, in *2023 IEEE International Conference on Robotics and Automation (ICRA)* (IEEE, 2023), pp. 12583–12589.

41. W. Yuan, C. Zhu, A. Owens, M. A. Srinivasan, E. H. Adelson, Shape-independent hardness estimation using deep learning and a gelsight tactile sensor, in *2017 IEEE International Conference on Robotics and Automation (ICRA)* (IEEE, 2017), pp. 951–958.
